# Measurement of Phenolic Environmental Estrogens in Women with Uterine Leiomyoma

**DOI:** 10.1371/journal.pone.0079838

**Published:** 2013-11-08

**Authors:** Yang Shen, Qian Xu, Mulan Ren, Xu Feng, Yunlang Cai, Yongxing Gao

**Affiliations:** 1 Department of Obstetrics and Gynecology, Zhongda Hospital, School of Medicine, Southeast University, Nanjing, China; 2 School of Public Health, Southeast University, Nanjing, China; 3 Ministry of Education Key Laboratory of Environmental Medicine Engineering, Southeast University, Nanjing, China; University of Kentucky, United States of America

## Abstract

**Objectives:**

To investigate the effect of phenolic environmental estrogens on uterine leiomyoma from the perspective of clinical epidemiology.

**Methods:**

Urine and blood samples were collected from Han women with uterine leiomyoma and women without uterine leiomyoma, living in Nanjing, China, between September 2011 and February 2013. A total of 156 urine samples and 214 blood samples were collected from the uterine leiomyoma group and 106 urine samples and 126 blood plasma samples from the control group. Bisphenol A (BPA), nonylphenol (NP) and octylphenol (OP) concentrations were determined by solid-phase extraction (SPE) coupled with liquid chromatography-tandem mass spectrometry (HPLC-MS/MS).

**Results:**

Phenolic environmental estrogens in the uterine leiomyoma and control groups were compared based on: gravida>3 and gravida ≤ 3. In participants with gravida>3, urine OP concentration was significantly (*P*<0.05) higher in the uterine leiomyoma group than in the control group. In participants with gravida ≤ 3, urine NP concentration was significantly (*P*<0.05) higher in the uterine leiomyoma group compared to controls. Despite obstetric history, urine BPA mean exposure concentration was significantly (*P*<0.05) different between uterine leiomyoma group and control group. The urine BPA concentration was not significantly (*P*>0.05) different between gravida>3 and gravida ≤ 3 patients. There was no significant (*P*>0.05) difference in plasma concentrations of BPA, OP and NP between the leiomyoma group and control group. Mean exposure concentration and range of distribution of BPA, OP and NP plasma concentration differed between the uterine leiomyoma and control group.

**Conclusion:**

Exposure level of phenolic environmental estrogens in human was related with leiomyoma tumorigenesis.

## Introduction

Uterine leiomyoma is one of the most common types of benign tumors affecting women aged 30 to 50 years. The incidence of uterine leiomyoma can be as high as 20–50%. It has been reported that the incidence of uterine leiomyoma in women following autopsy is higher than 77%, whilst the incidence of asymptomatic uterine leiomyoma is 50% [1]. As the incidence of uterine leiomyoma is increasing, attention must be paid to the prevention uterine leiomyoma. Currently, the causes of uterine leiomyoma remain poorly defined. Many gynecological researchers support the hypothesis that uterine leiomyoma is related to the levels of estrogen [2]. With the increase of environmental estrogen pollution, the concentration of bisphenol A (BPA), nonylphenol (NP) and octylphenol (OP) has become the primary indicator of phenolic environmental estrogens [3]. The current study establishes a method for the determination of BPA, OP and NP in human blood and urine samples based on solid-phase extraction coupled with liquid chromatography-tandem mass spectrometry (HPLC-MS/MS). In addition, this study analyzes the levels of exposure to BPA, OP and NP in relation to uterine leiomyoma tumorigenesis status.

## Materials and Methods

### Participant selection

All participants met the following standards: 30 to 50 years of age, premenopausal Han women; 15 years of continuous residency in the Nanjing province, or more than 30 years accumulated residency in the Nanjing province and less than 5 years outside the Nanjing province. The participants in the uterine leiomyoma group were selected from inpatients of the Obstetric Department of Zhongda Hospital affiliated with South-Eastern China University, Nanjing, China, whose uterine leiomyoma diseases were diagnosed by two independent doctors based on medical history, gynecologial examination, imaging examination (tumor diameter ≥ 5 cm) and postoperative pathological examination in the Obstetric Department of Zhongda Hospital affiliated with South-Eastern China University, Nanjing, China between September 2011 and February 2013. The health condition of participants in the control group was confirmed by the Obstetric Department of Zhongda Hospital or by physical imaging examination. It was ensured that participants did not use hormone drugs in the three months prior to the study, had no other reproductive system-related tumors, and did not have any estrogen-dependent diseases (malignant tumors, endocrine system diseases, etc.).

### Urine and blood sampling

The study protocol was reviewed and approved by the Ethics Committee of Zhongda Hospital affiliated with the South-Eastern University, Nanjing, China. All participants including patients with uterine leiomyoma signed an informed consent form. Participants provided a urine sample (8 mL) into glass tubes. Urine samples were stored at −20°C. Participants provided peripheral blood samples (5 mL) which were collected in anticoagulant tubes and stored at −4°C. In total, 312 urine samples were collected, including 156 from the uterine leiomyoma group and 116 from the control group. In total 340 blood plasma samples were collected, including 214 from the uterine leiomyoma group and 126 from the control group).

### Reagents and instruments

BPA, NP and OP were purchased from Aldrich-Sigma, St. Louis, MO, USA. OAS1S HLB solid-phase extraction cartridge was purchased from Waters, USA. The analytical balance (AT-250) was purchased from Mettler, Switzerland. The gas blowing concentrator was purchased from EYELA, USA. Solid-phase extractor, the Agilent 1100 series LC system, Agilent 6410 series spectrometer system and Agilent XDB-C18 (1.7 µm, 2.1×100 mm) were purchased from Sigma, USA.

### Standard solution preparation and sample pre-treatment

The standard solution was prepared as follows: standards of BPA, OP and NP (10 mg) were weighed into volumetric flasks, and dissolved in methanol to 100 mL resulting in 100 µg mL^−1^ standard stock solution. The stock solution was stored at −4°C.

The internal standard solution was prepared as followed. A (TBBPA), d_8_-NP, and d_16_-BPA with determined quantities were respectively weighed and dissolved in methanol, resulting in 100 µg mL^−1^ internal standard stock solution. The A (TBBPA) and d_8_-NP stock solution were combined with methanol resulting in a 1 µg mL^−1^ mixed internal standard solution for urine analysis. The d_16_-BPA and d_8_-NP stock solution with defined amounts were combined with methanol resulting in a 1 µg mL^−1^ mixed internal standard solution for blood analysis.

Samples were pre-treated according to the following protocol: urine samples (2.0 mL) or blood samples (0.5 mL) were placed into 10 mL glass centrifuge tubes. A total of 50 µL of mixed internal standard solution was added and the samples vortexed for 30 s. Blood samples were used for solid-phase extraction. Urine samples were successively added along with 200 µL acetic acid-sodium acetate buffer (pH = 5.5), 20 µL β-glucose anhydride and sulfatase for 3 h-enzymatic hydrolysis in a water bath at 37°C, followed by centrifugation at 3000 rpm. After 10 min centrifugation, the supernatant was collected for solid-phase extraction.

Phenolic environmental estrogens were extracted from samples by the solid-phase extraction method. The C18 SPE solid-phase extraction cartridge was activated by successively adding 1 ml dichloromethane, 3 ml methanol and 4 ml water. Defined quantities of prepared urine or blood samples were passed through the activated cartridge at an appropriate flow rate, followed by leaching with 1 ml pure water and 1 mL 70% methanol. The residual solution was removed by vacuum pump. The cartridge was eluted with 3 mL methanol and 1 ml dichloromethane. The eluent was evaporated to dry at 40°C under a stream of nitrogen, re-dissolved in the mobile phase and consequently used for HPLC analysis.

### HPLC-MS/MS conditions

The HPLC analysis was conducted on an Agilent XDB-C_18_ (50 mm×4.6 mm, 1.8 µm) chromatographic column at 30°C with an injection volume of 10 µL. The mobile phase consisted of methanol, water and 0.01 mol L^−1^ ammonium acetate (v/v). The mobile phase was delivered at a flow rate of 0.5 ml min^−1^. The system was run in a linear gradient: methanol flow increased from 10% to 70% in 0–3.0 min; methanol flow increased from 70% to 98% in 3–4 min; methanol flow was then held at 98% for 4–7.5 min then decreased to 10% after 7.5–8 min and stopped at 6 min.

Mass spectrometry equipped with an electrospray interface (ESI) and multiple reactions monitor (MRM) was operated in negative-ion scan model for this analysis. The MS parameters were set as followed: spray voltage 3.848 kV, Sheath Gas Pressure 35 psi, gas flow 10.0 L min^−1^, detector voltage 500 V, and ion source temperature 350°C.

### Statistical analysis

All statistical analysis was performed by SPSS 13.0 (SPSS Inc., Chicago, IL, USA).

The Student's t test was used to compare the ages of participants and uterine leiomyoma and the healthy control group. The Spearman's test was used to analyze the correlation between age and the obstetric history of participants.

## Results

### Age and obstetric history of participants

The age of participants was significantly different between the uterine leiomyoma and control group (*P*<0.05). In terms of individuals that participated in the urine sampling, age was significantly correlated with obstetric history (*r* = 0.563, *P*<0.05). For individuals that participated in blood sampling, age was also significantly correlated with obstetric history (*r* = 0.403, *P*<0.05). Consequently, participants were divided into two groups based on obstetric history: participants with gravida > 3 and participants with gravida ≤ 3. For each group, the rank sum test was used to test if the concentrations of three phenolic environmental estrogens in urine or blood differed between the uterine leiomyoma and control group.

### Phenolic environmental estrogens in human urine

The raw result of phenolic environmental estrogen in urine samples of the uterine leiomyoma and control group, detected by HPLC-MS/MS, included a large amount of data. Consequently, the concentration distribution of the three environmental estrogens was statistically analyzed and is summarized in Table 1, 2 and 3. The concentration of the three phenolic environmental estrogens appeared to be larger in the uterine leiomyoma group compared to the control group (Table 1). In participants with gravida > 3, the urine OP concentration was significantly higher in the uterine leiomyoma group than in control group (*P*<0.05). In participants with gravida ≤ 3, the urine NP concentration was significantly higher in the uterine leiomyoma group than that of the control group (*P*<0.05). The urine BPA concentration was not significantly different between participants with gravida ≤ 3 and those with gravida > 3 (*P*>0.05). Regardless of obstetric history, the mean exposure concentration of BPA, range of BPA distribution and number of participants with detectable levels of BPA differed between the uterine leiomyoma and control group.

**Table 1:** Concentrations of urine environmental estrogens (ng mL^−1^) in participants of uterine leiomyoma and control group (mean±SD, n = 156).

Compounds	Control group	Uterine leiomyoma group
BPA	11.788±1.738	17.586±2.329
OP	0.017±0.019	0.118±0.591(1)
NP	1.636±3.197	3.191±9.145(2)

(1)Participants with gravida > 3,compared with control group,*P*<0.05);(2)Participants with gravida ≤ 3,compared with control group,*P*<0.05).

**Table 2:** Detection of urine BPA, NP and OP in the uterine leiomyoma group (n = 98).

Compounds	BPA	OP	NP
Number of Samples	76	4	94
Detection rate	77.6%	4.08%	95.9%
Range (ng.mL^−1^)	1.06–59.6	1.2–4.34	0.72–37.6
Concentration (mean±SD) (ng.mL^−1^)	13.9±12.7	2.77±2.22	4.09±5.51

**Table 3:** Detection of urine BPA, NP, and OP in control group (n = 58).

Compounds	BPA	OP	NP
Number of participants	44	0	48
Detection rate	75.8%	0	82.8%
Range (ng.mL^−1^)	1.17–57.4	–	0.71–12.6
Concentration (mean±SD) (ng.mL^−1^)	8.50±12.2	–	3.84±3.90

### Phenolic environmental estrogens in human blood

The raw result of phenolic environmental estrogen in blood samples of the uterine leiomyoma and control group detected by HPLC-MS/MS included a large amount of data. Therefore, the distribution of concentrations of three environmental estrogens in human blood samples was statistically calculated and is summarized in Table 4, 5 and 6. The plasma BPA, OP, and NP concentrations were not significantly different between gravida ≤ 3 participants and gravida > 3 participants (*P*>0.05). However, regardless of obstetric history, the mean exposure concentration and range of distribution of BPA, OP, and NP concentration were significantly different between the uterine leiomyoma and control group (*P*<0.05).

**Table 4:** Blood plasma concentrations of environmental estrogens (ng mL^−1^) in uterine leiomyoma and controls (mean±SD, n = 340).

Compounds	Control group	Uterine leiomyoma group
BPA	7.062±8.840	5.046±5.911
OP	0.997±0.445	0.897±0.322
NP	17.074±12.151	13.647±12.646

**Table 5:** Detection of blood plasma BPA, NP and OP in the uterine leiomyoma group (n = 214).

Compounds	BPA	OP	NP
Number of participants	210	180	214
Detection rate	98.1%	84.1%	100%
Range (ng mL^−1^)	0.85–35.5	0.60–2.53	2.15–65.6
Concentration (ng.mL^−1^)	4.80±5.51	0.95±0.32	13.3±12.3

**Table 6:** Detection of blood plasma BPA, NP and OP in the control group (n = 126).

Compounds	BPA	OP	NP
Number of participants	124	126	122
Detection rate	98.4%	100%	96.8%
Range (ng.mL^−1^)	0.97–62.8	0.61–3.90	1.60–63.2
Concentration (mean±SD) (ng.mL^−1^)	7.53±9.44	1.01±0.48	18.7±12.3

## Discussion

Currently, the causes of uterine leiomyoma remain unknown. Many clinical observations and experimental studies have reported that uterine fibroids are estrogen-dependent [2]. Uterine fibroids may result from long-term stimulation of high levels of estrogens, in particular, environmental estrogens that enter the body via food intake and daily exposure [4].

Phenolic environmental estrogens include BPA, OP and NP. These phenolic environmental estrogens are produced worldwide, commonly used and thus easily exposed to humans. Phenolic environmental estrogens can disturb human endocrine function and be detrimental human health. Phenolic environmental estrogens can mimic or disturb endogenous estrogen by binding to the estrogen receptor or affecting estrogen cell signaling transduction by acting as an estrogen-like effect [5]–[6]. Uterine leiomyoma is sensitive to ovarian hormones and consequently is a likely target of environmental estrogens. This study investigated the relationship between environmental estrogens and uterine leiomyoma from the perspective of environmental toxicology by quantative determination of phenolic environmental estrogens in humans.

### Quantitative determination of individual exposure to phenolic environmental estrogens

Exposure concentration of environmental hazardous substances has previously been determined by detecting the actual concentrations of these substances in the environment, or by monitoring individual exposure. The concentration detected by the former method was external exposure dose compared to that of individual and internal exposure dose. Individual exposure monitoring quantitatively determines hazardous substance exposure and hazardous metabolic products in the biological materials of the body via biomonitoring. Such monitoring takes into account individual differences of exposure to hazardous substances. The results of the present study support reports of a correlation between disease and exposure to hazardous substances [7]. Consequently, an individual exposure method was employed in the current study to establish a method of determining BPA, OP and UP concentrations in human biological samples, thus providing relatively accurate results for the assessment of exposure levels of the three estrogens. This study highlights the importance of human epidemiological investigation and endocrine system-related diseases caused by the environment.

Human biological samples generally include blood, urine, saliva, latex and seminal fluid. In particular, blood and urine samples consist of complex matrix and have large individual differences. Consequently, in the current study, requirements for sample pre-treatment and analytical instruments for quantitative determinations were high.

Blood and urine samples analyzed in the current study were often pre-treated by liquid-liquid extraction, solid-phase extraction, and/or stir bar solid-phase extraction as previously described [8]–[9]. Compared with the other methods, solid phase extraction uses a smaller amount of organic solvent, has higher repeatability and is easier to automatic operation. Consequently, solid phase extraction is widely used for pre-treatment of samples with a complex matrix. The phenolic environmental estrogens in blood and urine are predominantly determined by HPLC, gas chromatography/mass spectrometry, HPLC-MS/MS and immunoassay [10]–[13]. Compared with other methods, HPLC-MS/MS is a quantitative and qualitative combined method. The HPLC-MS/MS method is highly efficient in terms of separation ability and is suitable to separate compounds with high boiling point, non-volatile and thermal instability. Therefore, HPLC-MS/MS is often used for the analyses of environmental estrogens in biological samples.

The current study establishes a quantitative determination method of BPA, OP and UP concentrations in human blood and urine samples based on SPE coupled with HPLC-MS/MS. The method was of low detection limit, high sensitivity and high repeatability, meeting the requirements of trace analysis of biological samples.

### Relationship between of phenolic environmental estrogens and uterine leiomyoma

Phenolic environmental estrogens enter the body through food intake and are secreted with stool and urine. The phenolic estrogen metabolic products in urine consist primarily of a conjugate of a conjugate of glucosides and sulphates [9]. The present study establishes a method to not only detect three phenolic environmental estrogens in human urine, but to also analyze the three phenolic environmental estrogens exposure levels. As the concentration of substances in human blood can reflect the status of their target organ in the body, this study also investigated the concentration of phenolic environmental estrogens in human blood samples to determine the exposure levels of BPA, OP and NP.

In the current study, compared with the control group, the uterine leiomyoma group showed significantly different urine OP concentrations, particularly in patients with gravida > 3, and significantly different urine NP concentrations in participants with gravida ≤ 3. Regardless of obstetric history, the urine BPA concentration was not significantly different between the uterine leiomyoma and control group. The number of participants with detectable levels of BPA, mean exposure concentrations of BPA and a range of urine distribution of BPA concentrations differed between the uterine leiomyoma and the control group. There was no significant difference in terms of plasma concentration of the three estrogens between the uterine leiomyoma group and the control group. The mean exposure concentration and range of plasma distribution of the three estrogens, however, were significantly different between the uterine leiomyoma and the control group. These results differ to previous studies reporting the relationship between exposure concentration of environmental estrogens in human and estrogen-dependent tumors affecting women. The results of the current study were unexpected. This may be as a result of analyzing blood and urine samples from different participants. Furthermore, participants in the control group were assured not to have uterine leiomyoma nor estrogen-related diseases. The possibility that some patients in the control group may have estrogen-related diseases can not be ruled out and is thus a limitation of the current study. The current study showed that estrogen exposure levels, the number of participants with detectable levels of estrogens, and the distribution range of estrogen concentrations differed between the uterine leiomyoma and control group, providing valuable information for future studies.

In summary, this study provides an appropriate standard for participant selection in future studies based on an established quantitative method. Future studies may clarify the relationship between exposure levels of phenolic environmental estrogen in human uterine leiomyoma tumorigenesis. In addition, results of future investigations may not only assist in lowering the incidence of uterine leiomyoma, but may also assist in the development of more comprehensive treatments for uterine leiomyoma diseases.

## References

[1] Duhan N, Sirohiwal D (2010) Uterine myomas revisited. Eur J Obstet Gynecol Reprod Biol 152: 119–125. 20933150 10.1016/j.ejogrb.2010.05.010

[2] Islam MS, Protic O, Giannubilo SR, Toti P, Tranquilli AL, et al (2013) Uterine leiomyoma: available medical treatments and new possible therapeutic options. J Clin Endocrinol Metab 98: 921–934. 23393173 10.1210/jc.2012-3237

[3] Calafat AM, Ye X, Wong LY, Reidy JA, Needham LL (2008) Exposure of the U.S. population to bisphenol A and 4-tertiary-octylphenol: 2003-2004. Environ Health Perspect 116: 39–44. 18197297 10.1289/ehp.10753 PMC2199288

[4] Maruo T, Ohara N, Wang J, Matsuo H (2004) Sex steroidal regulation of uterine leiomyoma growth and apoptosis. Hum Reprod Update 10: 207–220. 15140868 10.1093/humupd/dmh019

[5] Yu L, Moore AB, Castro L, Gao X, Huynh HL, et al (2012) Estrogen Regulates MAPK-Related Genes through Genomic and Nongenomic Interactions between IGF-1-I Receptor Tyrosine Kinase and Estrogen Receptor-Alpha Signaling Pathways in Human Uterine Leiomyoma Cells. J Signal Transduct 2012: 204–236. 10.1155/2012/204236 PMC3474284 23094148

[6] Ishikawa H, Ishi K, Serna VA, Kakazu R, Bulun SE, et al (2010) Progesterone is essential for maintenance and growth of uterine leiomyoma. Endocrinology 151: 2433–2442. 20375184 10.1210/en.2009-1225 PMC2875812

[7] Dekant W, Völkel W (2008) Human exposure to bisphenol A by biomonitoring: Methods, results and assessment of environmental exposures. Toxicology and Applied Pharmacology 228: 114–134. 18207480 10.1016/j.taap.2007.12.008

[8] Calafat AM, Kuklenyik Z, Reidy JA, Caudill SP, Ekong J, et al (2005) Urinary Concentrations of Bisphenol A and 4-Nonylphenol in a Human Reference Population. Environmental health perspectives 113: 391–395. 15811827 10.1289/ehp.7534 PMC1278476

[9] Kawaguchi M, Sakui N, Okanouchi N, Ito N, Saito K, et al (2005) Stir bar sorptive extraction with in situ derivatization and thermal desorption-gas chromatography-mass spectrometry for measurement of phenolic xenoestrogens in human urine samples. Journal of Chromatography B 820: 49–57. 10.1016/j.jchromb.2005.03.019 15866492

[10] Kawaguchi M, Ito R, Hayatsu Y, Nakata H, Sakui N, et al (2006) Stir bar sorptive extraction with in situ de-conjugation and thermal desorption gas chromatography-mass spectrometry for measurement of 4-nonylphenol glucuronide in human urine sample. Journal of Pharmaceutical and Biomedical Analysis 40: 82–87. 16019181 10.1016/j.jpba.2005.05.024

[11] Kuklenyik Z, Ekong J, Cutchins CD, Needham LL, Calafat AM, et al (2003) Simultaneous Measurement of Urinary Bisphenol A and Alkylphenols by Automated Solid-Phase Extractive Derivatization Gas Chromatography/Mass Spectrometry Analytical Chemistry. 75: 6820–6825. 10.1021/ac0303158 14670041

[12] Mao LH, Sun CJ, Zhang H, Yongxin Li, Desheng Wu (2004) Determination of environmental estrogens in human urine by high performance liquid chromatography after fluorescent derivatization with p-nitrobenzoyl chloride. Analytica Chimica Acta 522: 241–246.

[13] Ye X, Kuklenyik Z, Needham LL, Calafat AM (2005) Automated On-Line Column-Switching HPLC-MS/MS Method with Peak Focusing for the Determination of Nine Environmental Phenols in Urine. Analytical Chemistry 77: 5407–5413. 16097788 10.1021/ac050390d

